# Ginsenoside Rb1 Induces Beta 3 Adrenergic Receptor–Dependent Lipolysis and Thermogenesis in 3T3-L1 Adipocytes and *db/db* Mice

**DOI:** 10.3389/fphar.2019.01154

**Published:** 2019-10-15

**Authors:** Seona Lim, Jinbong Park, Jae-Young Um

**Affiliations:** ^1^Department of Science in Korean Medicine, Graduate School, Kyung Hee University, Seoul, South Korea; ^2^Department of Pharmacology, College of Korean Medicine, Kyung Hee University, Seoul, South Korea; ^3^Basic Research Laboratory for Comorbidity Research and Comorbidity Research Institute, College of Korean Medicine, Kyung Hee University, Seoul, South Korea

**Keywords:** ginsenoside Rb1, obesity, non-shivering thermogenesis, lipolysis, beta 3 adrenergic receptor

## Abstract

Obesity is constantly rising into a major health threat worldwide. Activation of brown-like transdifferentiation of white adipocytes (browning) has been proposed as a promising molecular target for obesity treatment. In this study, we investigated the effect of ginsenoside Rb1 (Rb1), a saponin derived from *Panax ginseng* Meyer, on browning. We used 3T3-L1 murine adipocytes and leptin receptor mutated db/db mice. The lipid accumulation, AMP-activated protein kinase alpha (AMPKα)–related pathways, lipolytic and thermogenic factors were measured after Rb treatment in 3T3-L1 adipocytes. Body weight change and lipolysis–thermogenesis factors were investigated in Rb1-treated db/db mice. Beta 3 adrenergic receptor activation (β3AR) changes were measured in Rb1-treated 3T3-L1 cells with or without β3AR inhibitor L748337 co-treatment. As a result, Rb1 treatment decreased lipid droplet size in 3T3-L1 adipocytes. Rb1 also induced phosphorylations of AMPKα pathway and sirtuins. Moreover, lipases and thermogenic factors such as uncoupling protein 1 were increased by Rb1 treatment. Through these results, we could expect that the non-shivering thermogenesis program can be induced by Rb1. In db/db mice, 6-week injection of Rb1 resulted in decreased inguinal white adipose tissue (iWAT) weight associated with shrunken lipid droplets and increased lipolysis and thermogenesis. The thermogenic effect of Rb1 was possibly due to β3AR, as L748337 pre-treatment abolished the effect of Rb1. In conclusion, we suggest Rb1 as a potential lipolytic and thermogenic therapeutic agent which can be used for obesity treatment.

## Introduction

Obesity is rising as a major health issue worldwide, especially in developed countries. As it is a risk factor for type 2 diabetes mellitus (T2DM) and other chronic metabolic disorders, the interest in managing obesity is constantly growing (Hossain et al., 2007). There are currently five different drugs approved by the United States Food and Drug Administration: orlistat, lorcaserin, phentermine/topiramate, naltrexone/bupropion, and liraglutide (Daneschvar et al., 2016). These drugs indeed help people lose weight, but serious side effects, such as steatorrhea, headache, nausea, vomiting, etc., encourage the search for alternative treatments (Azhar et al., 2016).

Obesity is a condition defined where excess/abnormal fat accumulation impacts health. As they are the key players in energy homeostasis, the importance of adipose tissues cannot be neglected in the strategy for obesity treatment. Adipose tissues can be categorized into two subsets based on function and morphology: white adipose tissue (WAT) and brown adipose tissue (BAT). The function of WAT is to store the energy in the form of lipids (Luo et al., 2016), while BAT dissipates energy as heat by a process called non-shivering thermogenesis (Yao et al., 2011). Non-shivering thermogenesis is a defense mechanism to fight against cold through enhanced mitochondrial content and uncoupling protein 1 (UCP1). Since its identification in the 16^th^ century, intense research on BAT revealed its role in thermogenesis during hibernation (Smith, 1961), and it is now appreciated as the main organ for non-shivering thermogenesis (Virtanen, 2016).

Recently, apart from classical BAT, recruitable brown fat has risen into another potential strategy for obesity management (Harms and Seale, 2013). These brown-like white adipocytes, also called “beige” or “brite” adipocytes, mimic the role of brown adipocytes by expressing abundant mitochondria associated with high expression levels of UCP1 (Carobbio et al., 2019). Beige and brown adipocytes share numerous characteristics, from the UCP1-mediated thermogenesis to several differentiation factors such as peroxisome proliferator–activated receptor gamma (PPARγ) coactivator 1 alpha (PGC1α) and RD1-BF1-RIZ1 homologous domain containing 16 (PRDM16) (Castro et al., 2017). As various stimuli such as cold exposure, endogenous signals, as well as dietary factors and pharmacological agents can induce the transdifferentiation of white adipocytes into beige adipocytes (Azhar et al., 2016), the search for relatively safe natural products which can induce “browning” of white adipocytes is a promising, attractive target for obesity treatment.

**Figure 1:**
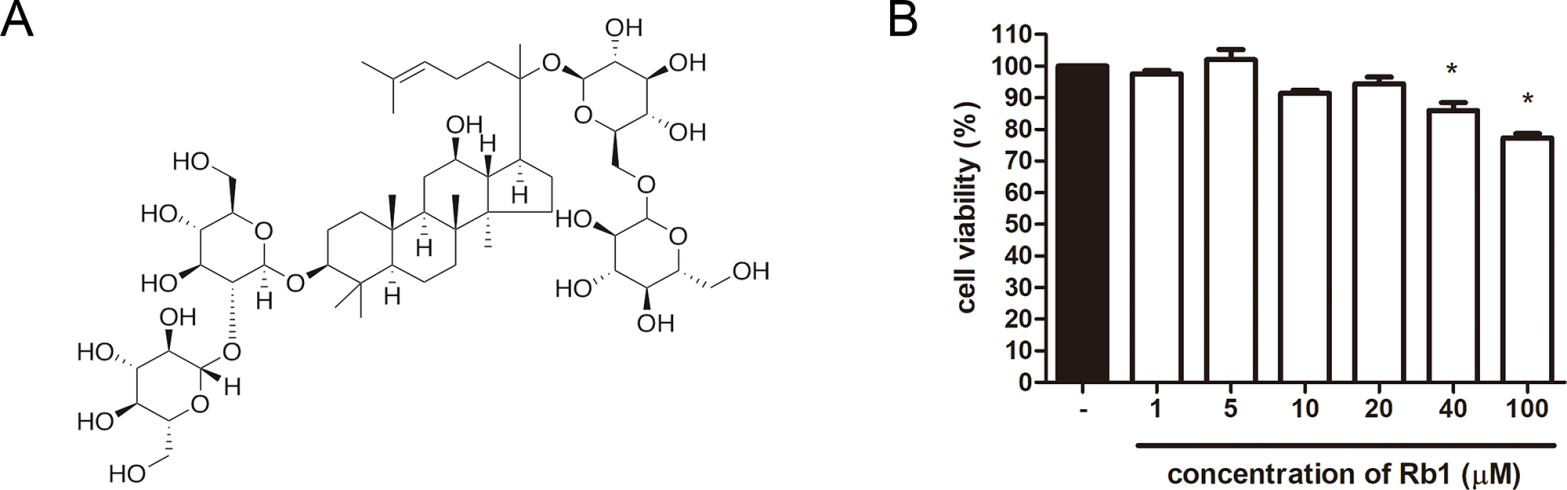
Chemical structure of Rb1 and cytotoxicity of Rb1 in 3T3-L1 adipocytes. **(A)** Chemical structure of Rb1 is shown. **(B)** An MTS assay was performed in order to evaluate the cytotoxicity of Rb1 on 3T3-L1 adipocytes. Data are expressed as mean ± standard error of the mean (S.E.M.) of three or more experiments. *p < 0.05 vs. untreated cells. Rb1, ginsenoside Rb1.

Ginsenoside Rb1 (C_54_H_92_O_23_, Rb1) (chemical structure shown in **Figure 1A**) is a triterpenoid saponin derived from the highly valued herb, *Panax ginseng* Meyer (*P. ginseng*). This herb has been used as a powerful tonic for qi and blood for over 50 centuries in traditional Chinese and Korean medicine and is also appreciated by the western countries as well (Zhou et al., 2019). Among the known saponins of *P. ginseng*, Rb1 is considered as one of the most abundant ingredients which may be responsible for the various biological functions of *P. ginseng* (Zhu et al., 2019). Several studies report the anti-adipogenic effect of Rb1 (Park et al., 2008; Xiong et al., 2010; Shen et al., 2013; Lin et al., 2014; Yu et al., 2015). In contrast, an early work by Shang et al. reported that Rb1 promotes adipogenesis by enhancing two major adipogenic factors, CCAAT/enhancer binding protein alpha (C/EBPα) and PPARγ (Shang et al., 2007). Furthermore, this was later supported by Mu’s study, as it showed that Rb1-induced increase of PPARγ and C/EBPα could have been a result of its browning effect in adipocytes. Mu’s team reported that Rb1 significantly increased the levels of UCP1, PGC1α, and PRDM16, thus leading to increased thermogenic capacity of 3T3-L1 adipocytes (Mu et al., 2015). However, although the browning effect of Rb1 has already been reported, its detailed mechanism still remains unknown to date. We hereby show that Rb1 treatment indeed resulted in browning of 3T3-L1 adipocytes, and this effect was due to regulation of beta 3 adrenergic receptor (β3AR)–mediated lipolysis induced by Rb1.

## Materials and Methods

### Chemical Reagents and Antibodies

Rb1 (>98%, ab142646) was purchased from Abcam (Cambridge, UK). 3-Isobutyl-1-methylxanthine (IBMX), dexamethasone (Dex), insulin, and Oil Red O powder were purchased from Sigma (St. Louis, MO, United States). L748337 was from Tocris Bioscience (Bristol, UK). Dulbecco’s Modified Eagle’s Medium (DMEM) and fetal bovine serum (FBS) were purchased from Corning (NY, United States). Antibodies for liver kinase B1 (LKB1) (3047S), pLKB1 (Ser428) (3482S), AMP-activated protein kinase alpha (AMPKα) (2532S), pAMPKα (Thr172) (2535S), acetyl-CoA carboxylase (ACC) (3676S), pACC (Ser79) (3661S), silent information regulator T1 (SIRT1) (8469S), SIRT3 (5490S), phospho-hormone sensitive lipase (pHSL) (Ser563) (4139S), phospho-PKA substrate (9624S), UCP1 (14670S), and β-actin (3700S) were purchased from Cell Signaling Technology (Beverly, MA, United States); the antibody for glyceraldehyde-3-phosphate dehydrogenase (GAPDH) (sc-32233) antibody was purchased from Santa Cruz Biotechnology (Santa Cruz, CA, United States); antibodies for PGC1α (ab54481), comparative gene identification 58 (CGI58) (ab59488), adipose triglyceride lipase (ATGL) (ab207799), HSL (ab45422), and β3AR (ab94506) were purchased from Abcam (Cambridge, UK); the antibody for PPARα (PA1-822A) was purchased from Thermo Fisher Scientific (Waltham, MA, USA).

### Cell Culture and Differentiation

3T3-L1 adipocytes from mouse embryo ﬁbroblasts cell lines were obtained from the American Type Culture Collection (Rockville, MD, USA), cultured, and differentiated into mature white adipocytes as previously described (Jung et al., 2018). Brieﬂy, cells were grown in DMEM containing 10% FBS and 100 units/ml of penicillin streptomycin solution at 37°C, in 5% CO_2_, at 95% humidity until conﬂuence. After 2 days from full conﬂuence (day 0), the cells were differentiated by a 48 h incubation in differentiation medium (DMEM plus 10% FBS containing 0.5 mM IBMX, 1 µM Dex, and 1 µg/ml insulin). At day 2, the cells were cultured in the maintenance medium (DMEM plus 10% FBS supplemented with 1 µg/ml insulin) and various concentrations of Rb1 (1, 5, 10 and 20 µM) for another 48 h followed by fresh maintenance medium.

Stromal vascular fraction (SVF) cells were isolated, cultured, and differentiated into mature beige adipocytes as previously described (Aune et al., 2013). Briefly, inguinal white adipose tissue (iWAT) was dissected from male 6-week-old C57BL/6J mice and digested in collagenase D and dispase II in 37°C with constant agitation at 150 rpm for 40–50 min. The cells were filtered using a cell strainer (50–70 μm diameter) and plated in 10 cm plates, and the red blood cells, immune cells, and other contaminants were removed by washing with PBS. Cells were grown in complete medium (DMEM containing 10% FBS and 100 units/ml of penicillin streptomycin solution) at 37°C, in 5% CO_2_, at 95% humidity until 95–97% conﬂuence (day 0). At day 0, the cells were differentiated by a 48 h incubation in differentiation medium consisting of maintenance medium (complete medium containing 5 µg/ml insulin and 1 nM T3) including 0.5 mM IBMX, 2 µg/ml Dex, and 125 µM indomethacin. After 48 h (day 2), the cells were cultured in maintenance medium with 0.5 µM rosiglitazone and 20 µM Rb1. After an additional 48 h (day 4), the medium was changed to fresh maintenance medium with 1 µM rosiglitazone for additional 2 days. At 6 days, cells are fully differentiated to mature beige adipocytes.

Human adipose tissue–derived mesenchymal stem cells (hAMSCs) (Cell Engineering for Origin, Seoul, Korea) were grown in DMEM containing 10% FBS and 100 units/ml of penicillin streptomycin solution at 37°C, in 5% CO_2_, at 95% humidity until conﬂuence. After 2 days from full conﬂuence (day 0), the cells were differentiated by a 72 h incubation in differentiation medium (DMEM plus 10% FBS containing 0.5 mM IBMX, 1 µM Dex 1 µM insulin, and 100 µM indomethacin). After 72 h (day 3), the cells were placed in fresh differentiation medium for an additional 3 days. At 6 days, the cells were cultured in the maintenance medium (consisting of DMEM with 10% FBS supplemented with 1 µM insulin) and 20 µM Rb1 for 72 h. From days 9 to 15, the maintenance medium was changed every 3 days.

Brown preadipocytes were obtained as described previously (Klein et al., 1999). Briefly, interscapular BAT was dissected from newborn FVB mice (age, post-natal day 1) and subjected to collagenase digestion for 30 min. The cells were filtered through a 100 μm strainer. The collected cells were centrifuged at 200 g for 5 min. Cells were seeded on 10 cm plates and grown in DMEM containing 10% FBS and 100 units/ml of penicillin streptomycin solution at 37°C, in 5% CO_2_, at 95% humidity. On day 2 after confluence (day 0), the cells were differentiated by a 48 h incubation in differentiation medium (DMEM plus 10% FBS containing 0.5 mM IBMX, 0.5 µM Dex, 20 nM insulin, 125 mM indomethacin, and 1 nM T3). At day 2, the cells were cultured in the maintenance medium (DMEM plus 10% FBS supplemented with 20 nM insulin and 1 nM T3) and 20 µM Rb1 for another 48 h followed by fresh maintenance medium.

### Cell Cytotoxicity Assay

Cell viability was measured with a Cell Proliferation MTS kit (Promega Co., Madison, WI, USA) as previously described (Jung et al., 2018). Brieﬂy, cells were seeded (2 × 10^4^ cells per well) on 96-well plates and incubated for 24 h, followed by incubation with various concentrations (1, 5, 10, 20, 40, and 100 μM in 3T3-L1 cells) of Rb1 in culture medium for an additional 48 h. The absorbance was measured at 490 nm in a VERSAmax microplate reader (Molecular Devices, Sunnyvale, CA, USA).

### Oil Red O Staining

Intracellular triglyceride (TG) accumulation was measured using the Oil Red O staining method as previously described (Jung et al., 2018). Photomicrograph images were obtained from a regular light microscope (Olympus, Tokyo, Japan), and absorbance was measured at 500 nm using a VERSAmax microplate reader (Molecular Devices, Sunnyvale, CA, USA).

### Protein Extraction and Western Blot Analysis

Western blot analyses were performed as previously described (Jung et al., 2018). In brief, homogenized tissues and harvested cells were lysed in lysis buffer (Cell Signaling Technology, Beverly, MA, United States), and protein concentration was determined using a protein assay reagent (Bio-Rad Laboratories, Hercules, CA, USA). Equal amounts of total protein were resolved by 8–12% sodium dodecyl sulfate–polyacrylamide gel electrophoresis and transferred to a polyvinylidene difluoride membrane. The membranes were incubated with the primary antibody at 4°C overnight and then incubated with a 1:10,000 dilution of the proper horseradish peroxidase (HRP)– conjugated secondary antibody (Jackson Immuno Research, West Grove, PA, USA) for 1 h at RT.

### Immunofluorescence Assay

Immunofluorescence (IF) analyses were performed as previously described (Jung et al., 2018). BODIPY 558/568 C12 (D3835) for staining lipid droplets was purchased from Thermo Fisher Scientific (Waltham, MA, USA). Images were acquired using a fluorescence microscope (Logos Biosystems, Anyang, Korea).

### Oxygen Consumption Rate Assay

Oxygen consumption rate (OCR) was measured by a Mito-ID Extracellular O_2_ sensor kit (OCR; ENZ-51045, ENZO Life Sciences, Farmingdale, NY, USA) according to the manufacturer’s instructions. Briefly, cells were prepared in 96-well black plates, and an O_2_ sensor probe (10 μl) was added into each well. After sealing the wells by adding two drops of provided oil, the absorbance was analyzed with a fluorescence plate reader.

### Free Fatty Acid Assay

Cellular free fatty acid (FFA) was measured by an EZ-Free Fatty Acid Assay Kit (DG-FFA100, Dogenbio, Seoul, Korea) according to the manufacturer’s instruction. Briefly, the FFA buffer was added to the prepared cells and reacted with the acyl-CoA synthesis for 30 min. After reaction, the reaction buffer was added and reacted at 37°C without light for 30 min. Then, the absorbance was read with a microplate reader.

### Cyclic Amp Assay

Cyclic AMP (cAMP) was measured using a cAMP ELISA Kit (STA-500, Cell Biolabs, Inc., San Diego, CA, USA) according to the manufacturer’s instruction.

### RNA Isolation and Real-Time Reverse Transcription Polymerase Chain Reaction

RNA extraction was using a GeneAll RiboEx Total RNA extraction kit (GeneAll Biotechnology, Seoul, Korea) according to the manufacturer’s instruction. cDNA synthesis was using a Maxime RT PreMix Kit (iNtRON Biotechnology, Seoul, Korea). Real-time reverse transcription polymerase chain reaction (RT-PCR) was performed with SYBR Green Power Master Mix (Applied Biosystems, Foster City, CA, USA) and the Step One Real-Time PCR System (Applied Biosystems, Foster City, CA, USA) according to the manufacturer’s instruction provided. The primers used in this study are provided in **Table 1**.

**Table 1:** Primer sequences used for real-time reverse transcription polymerase chain reaction (RT-PCR).

Primer, 5’–3’		
Target gene	Sense	Antisense
Atgl	ATATCCCACTTTAGCTCCAAGG	CAAGTTGTCTGAAATGCCGC
Cd137	TCTGTTGCTGGTCCTCAACTT	CTCCTACCTGGTCCTGAAAAC
Cpt1a	GACTCCGCTCGCTCATTCC	GACTGTGAACTGGAAGGCCA
Cpt1b	GCACACCAGGCAGTAGCTTT	CAGGAGTTGATTCCAGACAGGTA
Gapdh	AACTTTGGCATTGTGGAAGG	GGATGCAGGGATGATGTTCT
Prdm16	CCAAGGCAAGGGCGAAGAA	AGTCTGGTGGGATTGGAATGT
Tbx1	ATGATCTCCGCCGTGTCTAG	CGTGGGGAACATTCGTCTGCCTG
Tmem26	GAAATGCACCATGGAACCC	CGGTTCACATACCATGGATAA
Ucp1	AACTGTACAGCGGTCTGCCT	TAAGCCGGCTGAGATCTTGT
Adrb3	GGCCCTCTCTAGTTCCCAG	TAGCCATCAAACCTGTTGAGC

### Animal Experiment

Male 6-week-old *db/db* mice and age-matched wild-type (WT) C57BL6/J mice were purchased from Daehan Biolink Co. (Eumsung, Korea) and maintained for 1 week prior to the experiments, provided with a laboratory diet and water ad libitum. Healthy mice were randomly divided into three groups as follows (n = 5 per group): a WT group, a *db/db* group, and a *db/db* group administered with Rb1. The control groups (WT group, *db/db* group) were administered distilled water (i.p.), while the experiment group was administered Rb1 prepared in distilled water (10 mg/kg of body weight, i.p.) five times per week for 6 weeks. Body weight and food intake were measured three times per week. At the end of the experiment, the animals were anesthetized; tissues were collected and placed in a tube and then stored at −80°C.

### Hematoxylin and Eosin Staining

Hematoxylin and eosin (H&E) staining was done as previously described (Jung et al., 2018). Photographs were taken under a microscope, EVOS M7000 (Thermo Fisher Scientific, Waltham, MA, USA).

### Measurement of Body Fat by Dual-Energy X-Ray Absorptiometry Scan

A body fat scan was performed using dual-energy X-ray absorptiometry (DXA) with an InAlyzer instrument (Medikors, Seongnam, Korea) based on the manufacturer’s instructions as in a previous report (Lee et al., 2019).

### Statistical Analysis

Data were expressed as the means ± standard error of the mean (S.E.M.). Significant differences (*p* < 0.05) between groups were determined with the ANOVA test. All statistical analyzes were performed using SPSS statistical analysis software version 11.5 (SPSS Inc., Chicago, IL, USA).

## Results

### Rb1 Decreases the Size of Lipid Droplets in 3T3-L1 Adipocytes

**Figure 2:**
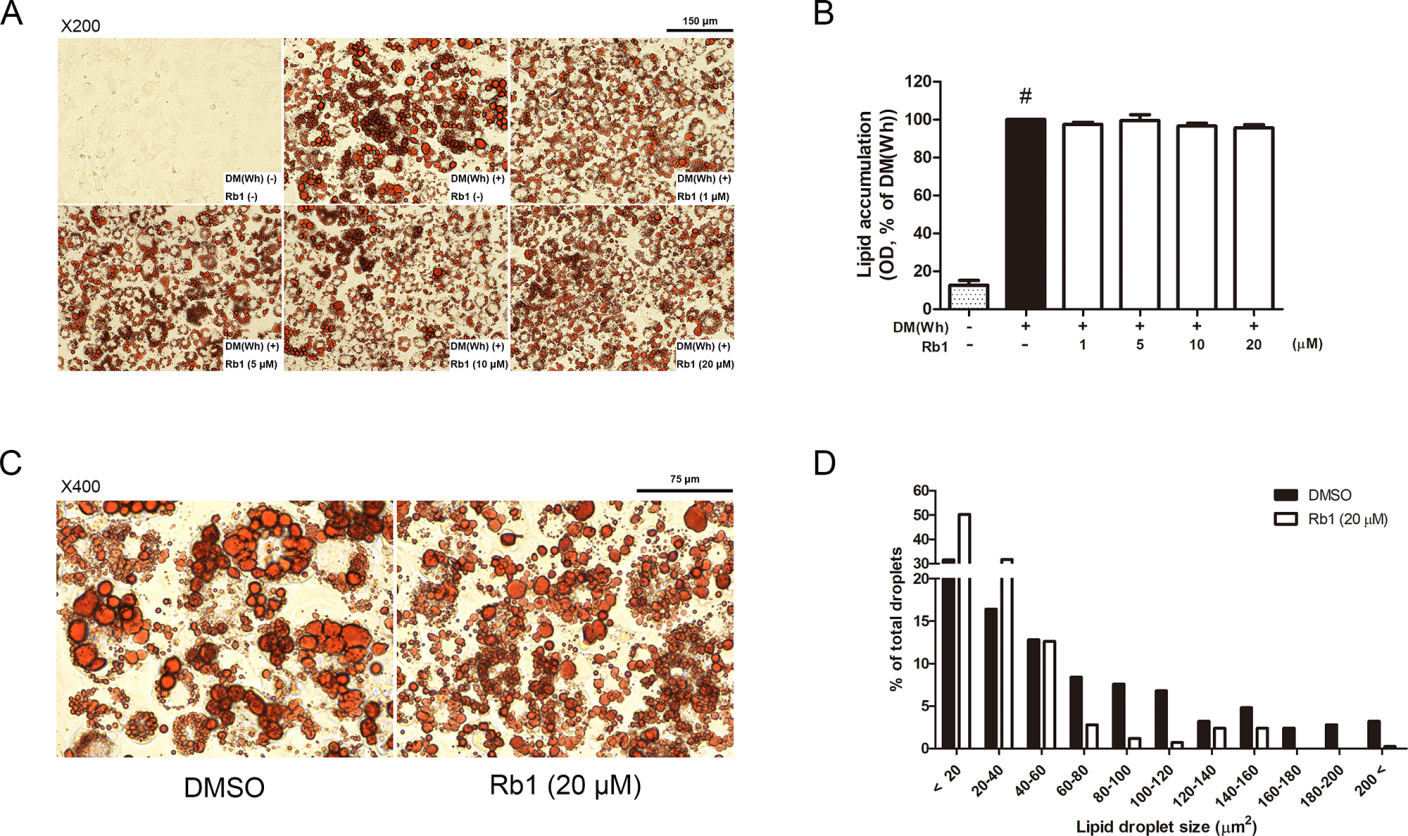
Effect of Rb1 on lipid accumulation in 3T3-L1 adipocytes. **(A)** Representative photomicrographs of Oil Red O staining in mature 3T3-L1 adipocytes (scale bar 150 μm). **(B)** Relative triglyceride (TG) level was quantified. **(C)** Effect of Rb1 (20 μM) on lipid droplet size was measured by Oil Red O staining (scale bar 75 μm). **(D)** Distribution of lipid droplet size was measured. Data are expressed as mean ± S.E.M. of three or more experiments. ^#^ *p* < 0.05 vs. un-differentiated preadipocytes. DM (Wh), white adipocyte differentiation media.

To evaluate the cytotoxicity of Rb1, an MTS assay was performed. As in **Figure 1B**, concentrations up to 20 μM did not affect cell viability of 3T3-L1 adipocytes. Then, an Oil Red O staining assay was conducted to assess the effect of Rb1 on lipid accumulation (**Figure 2A**). Rb1 treatment did not suppress nor increase TG accumulation in 3T3-L1 adipocytes (**Figure 2B**); however, the size of lipid droplets was significantly reduced in Rb1-treated cells when compared to differentiated adipocytes (**Figures 2C, D**). The portion of large lipid droplets (>160 µm^2^) was reduced from 8.4% to 0.4%, while the portion of small droplets (<20 µm^2^) was increased from 31.6% to 50.3%. The change in lipid droplet morphology suggests that Rb1 may have induced browning in 3T3-L1 adipocytes.

### Rb1 Increases Phosphorylation of LKB1–Ampkα–ACC Pathway and Expression of Sirtuin Proteins in 3T3-L1 Adipocytes

**Figure 3:**
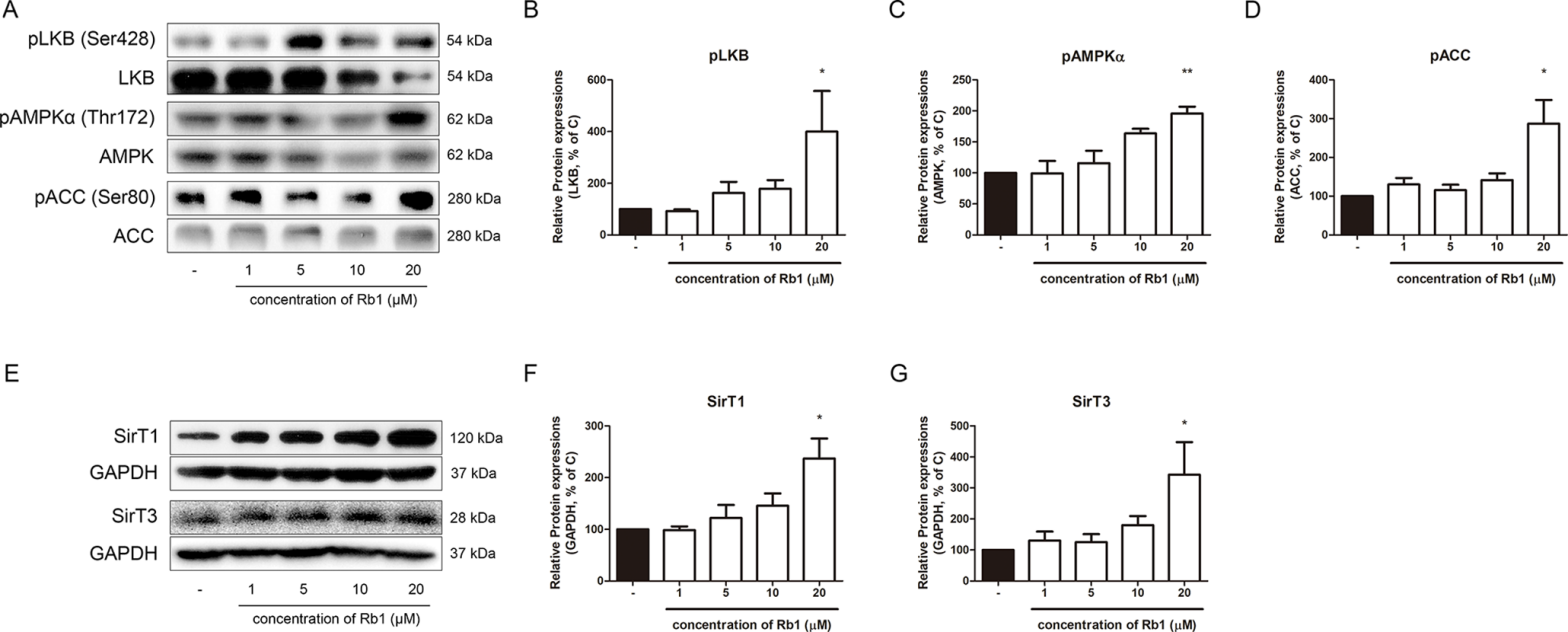
Effect of Rb1 on AMPK pathway in mature 3T3-L1 adipocytes. **(A)** Western blot assays were performed to measure the changes in pLKB1, LKB1, pAMPKα, AMPKα, pACC, and ACC. Relative expression levels of **(B)** pLKB1, **(C)** pAMPKα and **(D)** pACC were quantified. Expressions of pLKB1, pAMPKα and pACC were normalized against expressions of LKB1, AMPK, and ACC, respectively. **(E)** Western blot assays were performed to measure changes in SIRT1 and SIRT3. Relative expression levels of **(F)** SIRT1 and **(G)** SIRT3 were quantified. Expressions of SIRT1 and SIRT3 were normalized against GAPDH. Data are expressed as mean ± S.E.M. of three or more experiments. *p < 0.05 vs. vehicle-treated 3T3-L1 adipocytes; **p < 0.01 vs. vehicle-treated 3T3-L1 adipocytes.

AMPKα is a well-known sensor and regulator of energy metabolism (Steinberg and Carling, 2019). As shown in **Figures 3A–D**, Rb1 treatment upregulated the phosphorylation level of AMPKα in a concentration-dependent manner, showing that Rb1 can increase energy metabolism in 3T3-L1 adipocytes. Similar results were shown in the upstream and downstream factors of AMPKα, LKB1, and ACC, respectively.

After observing the effect of Rb1 on the AMPKα pathway, we then performed further experiments to verify whether Rb1 affects the levels of sirtuins. The sirtuins, consisting of seven homologs in mammals, are a family of NAD+-dependent histone/protein deacetylases (Michan and Sinclair, 2007). Among the seven sirtuins, SIRT1 and SIRT3 are closely related to the AMPKα function in lipid homeostasis (Ruderman et al., 2010; Shi et al., 2010). Our results showed that Rb1 also increased the levels of both sirtuins, but without statistical significance in SIRT3 (**Figures 3E–G**).

### Rb1 Induces Lipolysis in 3T3-L1 Adipocytes

**Figure 4:**
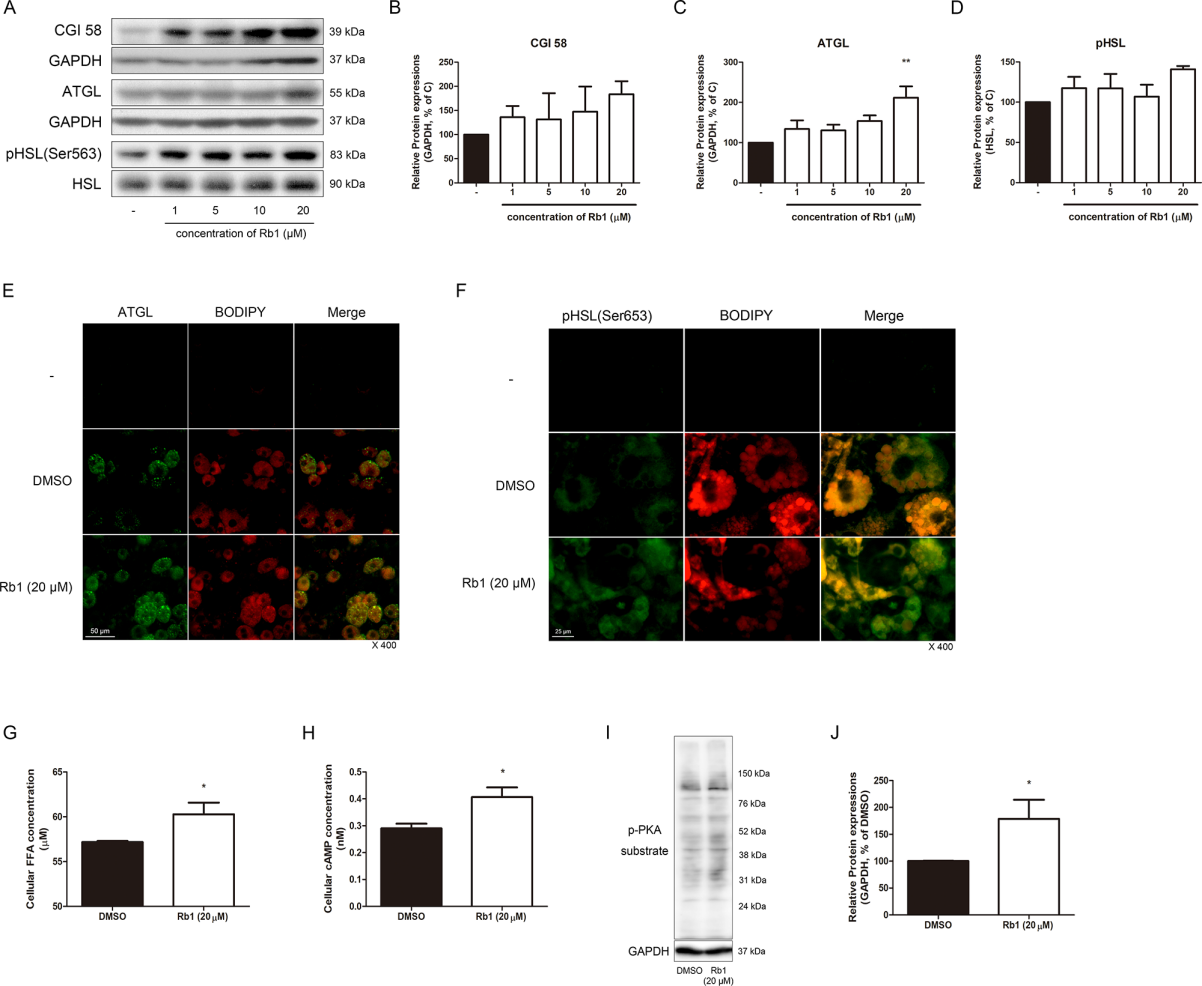
Effect of Rb1 on lipolysis in mature 3T3-L1 adipocytes. **(A)** Western blot assays were performed to measure the changes in CGI58, ATGL, and HSL. Relative expression levels of **(B)** CGI58, **(C)** ATGL, and **(D)** pHSL were quantified. Expressions of CGI58 and ATGL were normalized against GAPDH; expression of pHSL was normalized against HSL. **(E)** IF staining was performed to evaluate the expression level and pattern of ATGL and BODIPY (scale bar 50 μm). **(F)** Immunofluorescence (IF) staining was performed to evaluate the expression level and localization of pHSL (Ser563) and BODIPY (scale bar 25 μm). **(G)** Effect of Rb1 (20 μM) on cellular FFA concentration in mature 3T3-L1 adipocytes. **(H)** Effect of Rb1 (20 μM) on cellular cyclic AMP (cAMP) concentration in mature 3T3-L1 adipocytes. **(I)** Western blot assays were performed to measure the changes in p-PKA substrate. Relative expression level of **(J)** p-PKA substrate was quantified. Expression of p-PKA substrate was normalized against GAPDH. DMSO was used as vehicle. Data are expressed as mean ± S.E.M. of three or more experiments. *p < 0.05 vs. vehicle-treated 3T3-L1 adipocytes; **p < 0.01 vs. vehicle-treated 3T3-L1 adipocytes.

AMPKα, the energy metabolism sensor, also acts as a regulator of lipolysis by phosphorylating ATGL and HSL, the two major lipases working in the lipolytic process (Kim et al., 2016). Since we verified the effect of Rb1 on the AMPKα pathway and sirtuins, our next objective was to investigate its effect on lipolysis. CGI58, a critical regulator of ATGL, showed an increasing tendency in Rb1-treated adipocytes (**Figures 4A, B**). In addition, protein levels of ATGL was significantly increased by Rb1 treatment, while pHSL was not (**Figures 4A, C, D**). The lipolytic activation induced by Rb1 was re-confirmed when we performed an IF staining assay of ATGL and pHSL (**Figures 4E, F**).

Furthermore, intracellular FFA was increased by Rb1 treatment (*p* < 0.05), confirming its lipolytic effect (**Figure 4G**). As cAMP and its downstream target PKA are regulators of lipolysis, we then measured the change in these factors. Indeed, cAMP and PKA both were significantly induced in Rb1-treated 3T3-L1 cells (**Figures 4H, I, J**).

To confirm, we obtained SVF cells from iWAT of C57BL/6J mice and induced beige differentiation based on a previous report (Aune et al., 2013). Rb1 treatment in SVF cells also increased the mRNA expression of *Atgl*, the gene which transcripts ATGL (**Supplementary Figure S1**).

### Rb1 Increases Thermogenic Capacity in 3T3-L1 Adipocytes and Induces Browning

**Figure 5:**
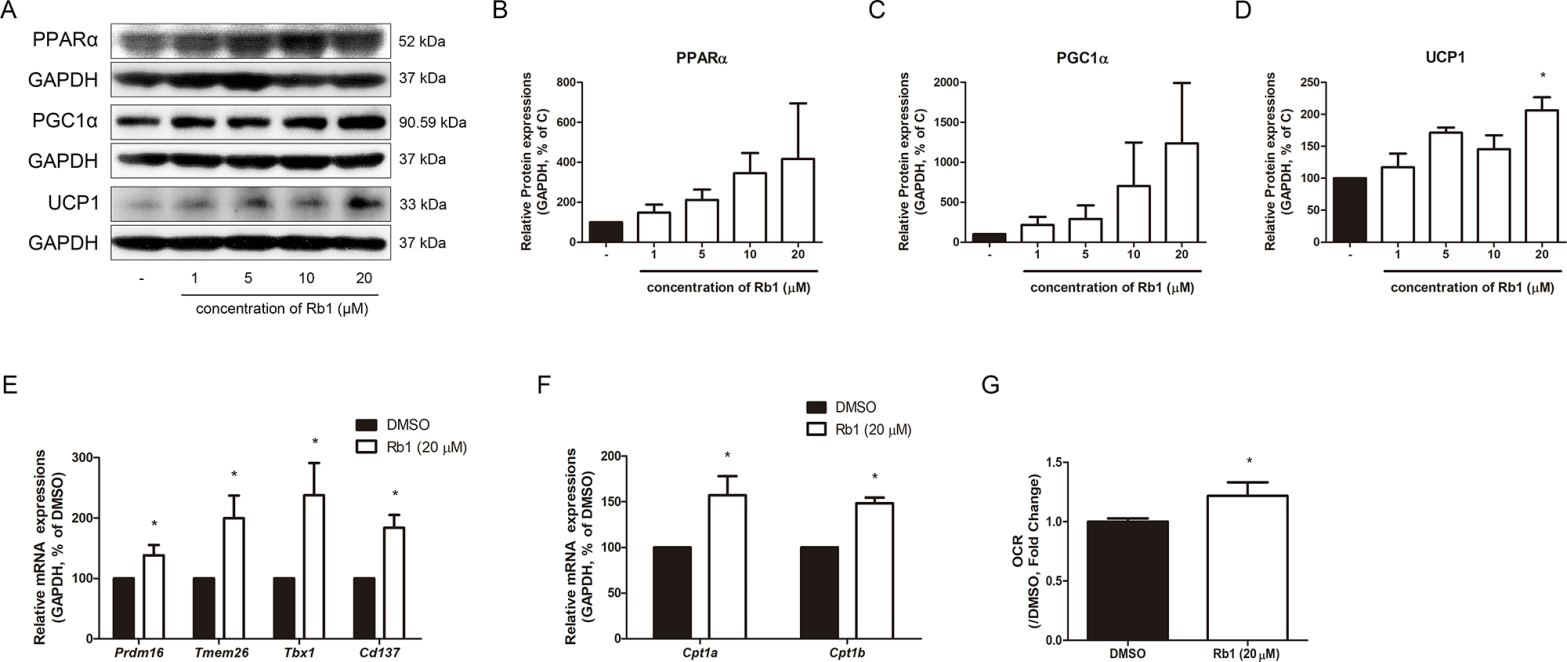
Effect of Rb1 on browning in mature 3T3-L1 adipocytes. **(A)** Western blot assays were performed to measure the changes of PPARα, PGC1α, and UCP1. Relative expression levels of **(B)** PPARα, **(C)** PGC1α, and **(D)** UCP1 were quantified. Expressions of PPARα, PGC1α, and UCP1 were normalized against GAPDH. **(E)** mRNA levels of beige-specific genes (*Prdm16, Tmem26, Tbx1 and Cd137*) and **(F)** mitochondrial beta oxidation–related genes (*Cpt1a* and *Cpt1b*) were analyzed by RT-PCR. Relative mRNA level of each gene was normalized against *Gapdh*. **(G)** Effect of Rb1 (20 μM) on OCR in mature adipocytes. DMSO was used as vehicle. Data are expressed as mean ± S.E.M. of three or more experiments. *p < 0.05 vs. vehicle-treated 3T3-L1 adipocytes.

Lipolysis is known as the pre-step of brown and beige adipocyte thermogenesis. Importantly, lipolysis plays a central role in the catabolic activity of BAT and WAT. FAs induce oxidative phosphorylation and provide fuel that supports UCP1-mediated respiration (Granneman et al., 2003). Lipolysis also provides ligands for PPARα, which acts crucially in the browning of WAT (Li et al., 2005). As shown in **Figures 5A, B**, Rb1-treated adipocytes showed increased levels of PPARα, implying the thermogenic triggering by Rb1. PGC1α, the major transcription factor of both brown and beige adipocytes (Inagaki et al., 2016), was also increased by Rb1 treatment (**Figures 5A, C**). The thermogenic capacity was determined by assessing the changes in UCP1 (**Figures 5A, D**). Rb1 treatment induced a 2.06-fold change in the protein expression of UCP1 when compared to vehicle-treated adipocytes. Similar results were retrieved from Rb1-treated SVF cells (**Supplementary Figure S1**) and hAMSCs (**Supplementary Figure S2**) as well. Further real-time RT-PCR assays showed increased beige-specific gene levels of *Prdm16*, *Tmem26*, *Tbx1*, and *CD137* in Rb1-treated cells (**Figure 5E**).

Upregulated factors of mitochondrial beta oxidation such as mRNA of *Cpt1a* and *Cpt1b* also indicated the Rb1-induced thermogenic capacity (**Figure 5F**). Since the thermogenic action of UCP1 requires alteration of oxidative levels, we evaluated the change in O_2_ consumption, which in turn was found to be increased by Rb1 treatment (**Figure 5G**).

### Rb1 Induces Browning in iWAT of *db/db* Mice

**Figure 6:**
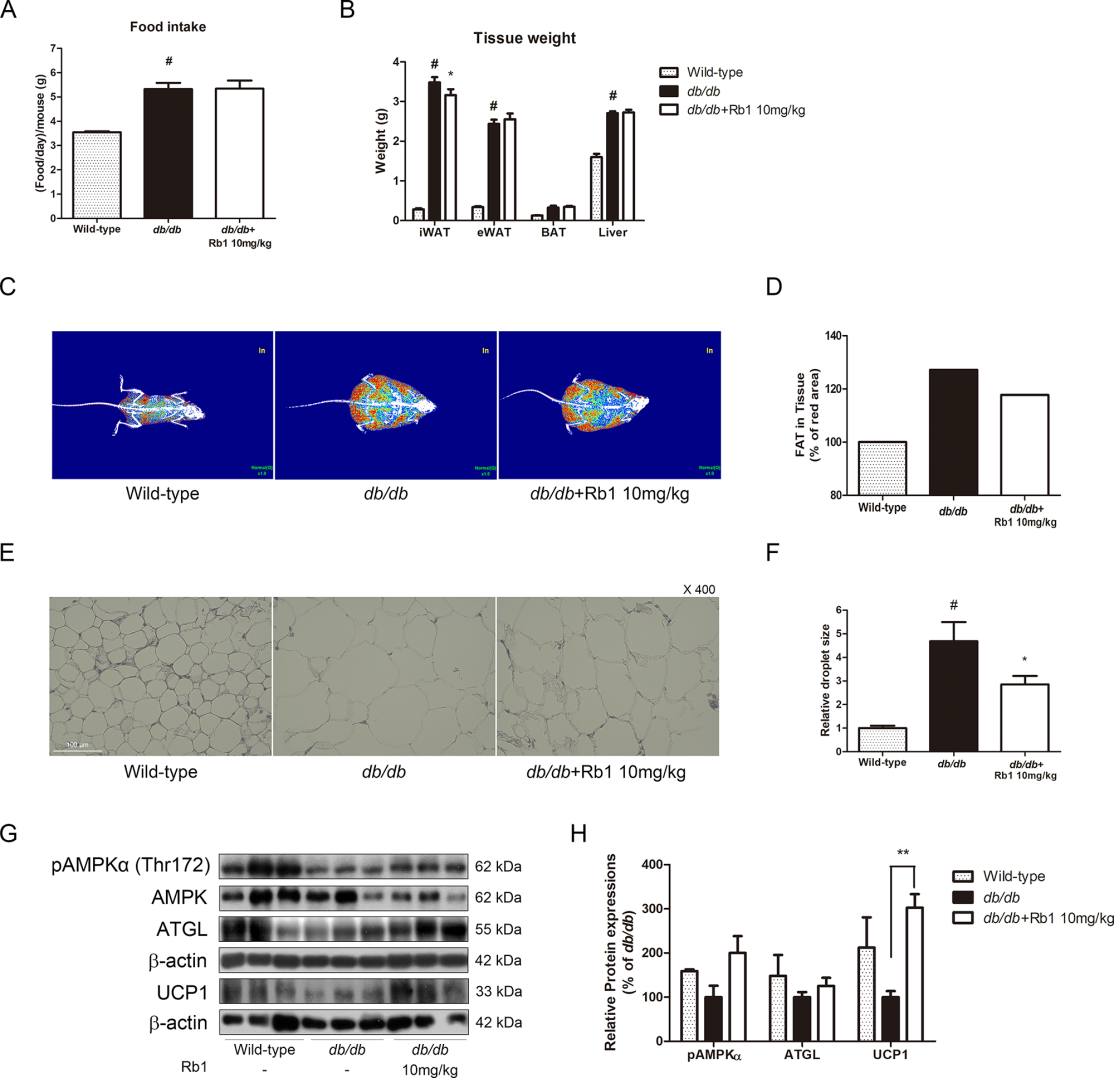
Effect of Rb1 on weight change, lipolysis, and thermogenesis in *db/db* mice. **(A)** Food intake of each group was measured. **(B)** Tissue weight of iWAT, eWAT, BAT, and liver of each group was measured. **(C)** Body fat was measured using DXA scan. Red signal indicates fat composition. **(D)** Relative red signal in DXA scan was measured. **(E)** H&E staining was performed to evaluate histological changes in iWAT of *db/db* mice (scale bar 100 μm). **(F)** Relative lipid droplet sizes in iWAT of each group were measured. **(G)** Western blot assays were performed to measure the changes of pAMPKα, AMPKα, ATGL, and UCP1. **(H)** Relative expression levels of pAMPKα, AMPKα, ATGL, and UCP1 were quantified. Expression of pAMPKα was normalized against AMPKα; expressions of ATGL and UCP1 were normalized against β-actin. PBS was used as vehicle. Data are expressed as mean ± S.E.M. of three or more experiments. ^#^ *p* < 0.05 vs. vehicle-treated wild-type C57BL6/J mice; *p < 0.05 vs. vehicle-treated *db/db* mice; **p < 0.01 vs. vehicle-treated *db/db* mice.

We then conducted an animal study to evaluate the effect of Rb1 *in vivo*. After feeding obese *db/db* mice with Rb1 (10 mg/kg/day) for 6 weeks, we observed unchanged food intake between vehicle-fed and Rb-fed *db/db* mice (**Figure 6A**). Surprisingly, not in accordance with the *in vitro* results, Rb1 did not affect body weight change in *db/db* mice (data not shown). However, a significant reduction in iWAT was shown in Rb1-treated mice, while epididymal white adipose tissue (eWAT), BAT, and liver tissue weight were not affected (**Figure 6B**). A DXA scan analysis showed decreased fat body mass in the Rb1-fed group compared to the vehicle-treated *db/db* group (**Figures 6C, D**). Next, we evaluated the Rb1-induced histological changes in iWAT. As a result, the average size of lipid droplets was increased by 4.68-fold in *db/db* mice compared to WT mice, and this was decreased down to 61% by Rb1 administration (**Figures 6E, F**).

Further western blot assays were performed to evaluate the effect of Rb1 on thermogenesis-related factors. As shown in **Figures 6G, H**, Rb1 treatment increased the protein expressions of pAMPKα, ATGL, and UCP1 in iWAT of mice, suggesting that a browning effect was induced by Rb1.

### Thermogenic Effect of Rb1 Is Dependent on β3AR Pathway

**Figure 7:**
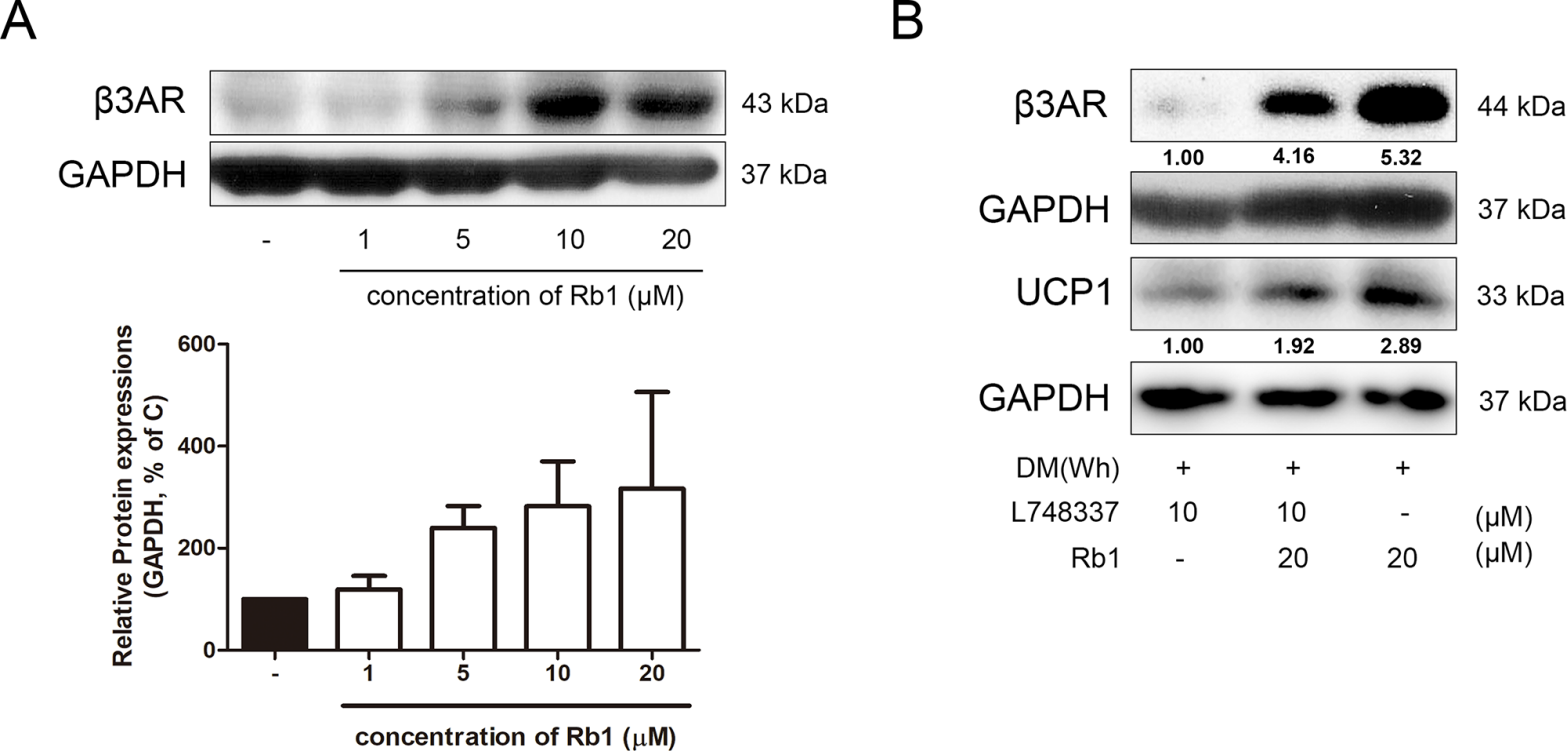
Effect of Rb1 on β3AR in mature 3T3-L1 adipocytes. **(A)** A western blot assay was performed to measure the change in β3AR. Expression of β3AR was normalized against GAPDH. **(B)** Changes in β3AR and UCP1 after β3AR inhibition were measured by western blot assays. Data are expressed as mean ± S.E.M. of three or more experiments.

As β3AR is considered as one of the highest upstream signals of non-shivering thermogenesis (Arch, 2002; Dulloo, 2011), we attempted to investigate whether Rb1 could regulate the expression of this receptor. As expected, Rb1 treatment in 3T3-L1 adipocytes resulted in a dose-dependent increase of β3AR expression (**Figure 7A**). Similar results were observed in Rb1-treated SVF cells and hAMSCs (**Supplementary Figures S1 and S2**). Furthermore, when L748337, a selective β3AR antagonist, was pre-treated in adipocytes, the effect of Rb1 on UCP1 induction was abolished, down to nearly 67% (**Figure 7B**), suggesting that the thermogenic effect of Rb1 is dependent on the β3AR signaling pathway, at least partially.

Since β3AR is closely related to the activation of classical brown fat as well, we attempted to figure out whether Rb1 treatment regulates thermogenesis in BAT. As in **Supplementary Figures S3A, B**, Rb1-treated *db/db* mice displayed a significantly higher level of β3AR in BAT. Consequently, lipolysis and non-shivering thermogenesis seemed to be induced, when confirmed by the protein levels of ATGL and UCP1. An *in vitro* study with primary cultured brown adipocytes showed similar results (**Supplementary Figure S3C**).

## Discussion

The negative impact of obesity on mankind health forces clinicians and researchers to seek a promising anti-obese strategy. In 2016, the World Health Organization (WHO) reported that around 2 billion adults were overweight, and 650 million obese (WHO, 2018). However, currently available strategies, mostly medications, display unwelcome side effects (Daneschvar et al., 2016). Thus, the task of safe and effective anti-obese agents is still an ongoing challenge. In this context, the potential of natural products, also proved by the steady growth of their market size (Brown, 2017), may give an advantage for the next promising strategy for obesity care.

There are some obvious clues to the anti-obese action of Rb1 from previously published literature. Park et al. reported that Rb1 and another saponin of *P. ginseng*, ginsenoside Rg1, suppressed TG accumulation *in vitro* (Park et al., 2008), while Xiong et al. showed that the anti-obese effect of Rb1 was effective *in vivo* as well (Xiong et al., 2010). Shen et al. suggested that AMPK was responsible for this effect (Shen et al., 2013), Lin and colleagues explained that it resulted from decreased appetite by Rb1-regulated neuropeptide Y (NPY) and peptide YY (PYY) (Lin et al., 2014), and Yu et al. reported that perilipin expression was the clue (Yu et al., 2015). Further evidence also supports the potential thermogenic effect of Rb1. Several studies have recently reported the browning or lipolytic effect of a wide variety of ginsenoside isoforms besides Rb1. Regarding ginsenoside Rg1, a quantitively important isoform (Lü et al., 2009), it is known to reduce TG accumulation in adipocytes (Park et al., 2008), suppress hepatic glucose production in HepG2 cells (Kim et al., 2010), and increase glucose uptake in muscle cells (Lee HM et al., 2012). Furthermore, Li et al. reported its beneficial effect in glucose metabolic disorder (Li et al., 2018), and Liu et al. showed the AMPK-mediated anti-obese effect of ginsenoside Rg1 (Liu et al., 2018). A recent study also reported the browning potential of ginsenoside Rg1 (Lee et al., 2018). Ginsenoside Rg3 also shows improvement in metabolic diseases *in vivo* and *in vitro* (Hwang et al., 2009; Kim SN et al., 2009; Lee et al., 2011; Lee et al., 2017; Zhang et al., 2017). Ginsenoside Rb2, another abundant isoform of ginsenosides, is reported to possess beneficial effects on lipid accumulation. Cholesterol and TG levels were attenuated by ginsenoside Rb2 treatment in 3T3-L1 adipocytes (Kim EJ et al., 2009), and similar results were shown in high-fat diet–fed obese mice (Dai et al., 2018). Moreover, significant activation of thermogenic factors in BAT and WAT were observed in ginsenoside Rb2–fed obese mice (Hong et al., 2019). Through these obvious hints from previous literature, we could expect the potential benefits from Rb1; however, to date, the exact cascade mechanism of the thermogenic effect of Rb1 still remains to be elucidated. Therefore, in this study, we aimed to investigate the detailed action mechanism of Rb1 on beige adipocyte recruitment and thermogenesis induction using *db/db* mice and 3T3-L1 adipocytes.

AMPK is a metabolism regulator protein consisting of three subunits: catalytic α, regulatory β, and γ (Hardie, 2018). As its name suggests, the elevation of AMP/ADP associated with reduced ATP triggers the activating phosphorylation of AMPK specifically at Thr172 of the α subunit (Oakhill et al., 2012). Once phosphorylated, AMPK regulates activation of metabolic proteins and transcription factors, leading to an energy production. Numerous nature-derived materials such as berberine (Kim WS et al., 2009), quercetin (Ahn et al., 2008), and resveratrol (Wang et al., 2015) are shown to induce AMPK activation and thus ameliorate metabolic diseases. Our results also suggest the possible use of Rb1 as an AMPKα-activating anti-obesity agent. Rb1 increased phosphorylation of not only AMPKα but also its upstream kinase LKB1 and downstream factor ACC. In addition, Rb1 increased the levels of SIRT1, which is known to facilitate metabolic AMPK action (Ruderman et al., 2010).

Non-shivering thermogenesis in BAT and WAT is a promising molecular target for obesity management. The recently discovered beige adipocytes within WAT differ from classic white adipocytes both by morphology and ability. By certain stimulation, such as cold exposure (Vitali et al., 2012; Wu et al., 2012) or pharmacological activation (Lee JY et al., 2012; Rachid et al., 2015), these beige adipocytes, which were normally acting as a storage unit for lipid, become capable of producing heat through mitochondrial UCP1 activation. Once activated in either BAT or WAT, the thermogenic action of mitochondria requires fuel: FAs. In order to supply FAs, the lipolysis signaling is activated within the adipocyte, and the lipases ATGL, HSL, and monoacylglycerol lipase (MG) subsequently process TG into FAs for β-oxidation (Steensels and Ersoy, 2019). In this study, we observed that Rb1 can increase ATGL, suggesting a possible role of Rb1 in induction of the lipolysis signaling for thermogenic actions.

β3ARs have a critical role in activation of non-shivering thermogenesis *via* PKA signaling, in both BAT and “beiged” WAT (Lowell and Spiegelman, 2000; Cao et al., 2001). Jimenez et al. showed that β3AR knocked-out mice displayed a low mRNA level of Ucp1 and failed to induce thermogenesis by cold stimuli (Jimenez et al., 2003). In accordance, β3AR activation by pharmacological agonists CL316,243 and isoproterenol results in enhanced UCP1 expression in BAT (Bachman et al., 2002) and higher thermogenic capacity in WAT (Wang et al., 2013). Nature-derived nutritional agents are also candidates for β3AR-activated thermogenesis as well. Ephedrine, an active compound of genus *Ephedra*, is shown to induce β3AR expression and glycerol release, which was potentiated with β3AR agonist BRL37344 and inhibited by β3AR antagonist SR59230A co-treatment (Cheng et al., 2001). Another team reported that cinnamon extract induces browning in 3T3-L1 adipocytes and WAT of high-fat diet–induced obese mice by activating β3AR (Kwan et al., 2017). Our results suggest another natural product which can induce browning by β3AR activation. Rb1 administration increased expressions of lipases and thermogenic factors including UCP1, and these effects were suppressed when a β3AR antagonist, L748337, was pre-treated. Although activation of β3AR may benefit metabolic diseases such as obesity, the risk of β3AR in cardiovascular diseases cannot be neglected (Cossu et al., 1997; Sears, 2002). However, various studies report that the effect of Rb1 does not harm but even possibly improves the cardiovascular system. Rb1 can benefit myocardial ischemia (Guan et al., 2002; Wang et al., 2008; Wu et al., 2011; Xia et al., 2011; Kong et al., 2015; Yan et al., 2015; Li et al., 2016; Yan et al., 2016; Cui et al., 2017; Zheng et al., 2017a), atherosclerosis/vascular dysfunctions (Qiao et al., 2017; Zhou et al., 2017; Zhang et al., 2018), or other related diseases (Jiang et al., 2007; Kong et al., 2010; Li et al., 2011; Li et al., 2012; Zhang et al., 2015; Zheng et al., 2017b), suggesting the potential safety of Rb1 in cardiovascular functions, despite its action on β3AR.

In our study, we have shown that Rb1 can decrease lipid accumulation *in vivo* and *in vitro* by inducing the lipolysis–thermogenesis cascade. This effect was probably due to activation of β3ARs, as β3AR inhibitor treatment decreased the thermogenic effect of Rb1. However, further studies are required to understand the whole-body significance of Rb1-activated β3AR, as our study mainly focused on the beige adipocyte recruitment in WAT. Because the mechanisms of WAT browning and BAT activation are both related in the β3AR-dependent thermogenesis, thus, to investigate the whole precise mechanism of the anti-obese effect of Rb1, relevant studies dealing with the role of BAT in the effect of Rb1 are necessary. Furthermore, as the non-adrenergic pathway is also capable of progressing lipolysis and thermogenesis (Braun et al., 2018), related investigation on Rb1 action has to be carried out as well.

Overall, our results demonstrate the effect of Rb1 on β3AR-dependent lipolysis and thermogenesis. Regarding the well-known clinically beneficial features of *P. ginseng* in traditional Korean medicine, we suggest Rb1 as a potentially safe and effective therapeutic agent for treatment of metabolic diseases.

## Data Availability Statement

The raw data supporting the conclusions of this manuscript will be made available by the authors, without undue reservation, to any qualified researcher.

## Ethics Statement

All animal experiments were performed according to the Guide for the Care and Use of Laboratory Animals and were approved by the Institutional Review Board of Kyung Hee University (confirmation number: KHUASP (SE)-13-012).

## Author Contributions

SL, JP and J-YU designed the protocol and prepared the manuscript; SL and JP performed the experiments; J-YU was in charge of the whole experiment conduction and proofreading of the manuscript. All authors approved the final version to be published.

## Funding

This study was supported by the National Research Foundation of Korea (NRF) grant funded by the Korea government (MSIP) (NRF-2015R1A4A1042399, 2018R1D1A1B07049882 and 2018R1A2A3075684).

## Conflict of Interest

The authors declare that the research was conducted in the absence of any commercial or financial relationships that could be construed as a potential conflict of interest.

## References

[B1] Ahn J. Lee H. Kim S. Park J. Ha T. (2008). The anti-obesity effect of quercetin is mediated by the AMPK and MAPK signaling pathways. Biophys. Res. Commun. 373, 545–549. 10.1016/j.bbrc.2008.06.077 18586010

[B2] Arch J. R. (2002). beta(3)-Adrenoceptor agonists: potential, pitfalls and progress. Eur. J. Pharmacol. 440, 99–107. 10.1016/S0014-2999(02)01421-8 12007528

[B3] Aune U. L. Ruiz L. Kajimura S. (2013). Isolation and differentiation of stromal vascular cells to beige/brite cells. J. Vis. Exp. 73, e50191. 10.3791/50191 PMC3641667 23568137

[B4] Azhar Y. Parmar A. Miller C. N. Samuels J. S. Rayalam S. (2016). Phytochemicals as novel agents for the induction of browning in white adipose tissue. Nutr. Metab. (Lond.) 13, 89. 10.1186/s12986-016-0150-6 27980598 PMC5135798

[B5] Bachman E. S. Dhillon H. Zhang C. Y. Cinti S. Bianco A. C. Kobilka B. K. (2002). betaAR signaling required for diet-induced thermogenesis and obesity resistance. Science 297, 843–845. 10.1126/science.1073160 12161655

[B6] Braun K. Oeckl J. Westermeier J. Li Y. Klingenspor M. (2018). Non-adrenergic control of lipolysis and thermogenesis in adipose tissues. J. Exp. Biol. 221, jeb165381. 10.1242/jeb.165381 29514884

[B7] Brown A. C. (2017). An overview of herb and dietary supplement efficacy, safety and government regulations in the United States with suggested improvements. Part 1 of 5 series. Food Chem. Toxicol. 107, 449–471. 10.1016/j.fct.2016.11.001 27818322

[B8] Cao W. Medvedev A. V. Daniel K. W. Collins S. (2001). beta-Adrenergic activation of p38 MAP kinase in adipocytes: cAMP induction of the uncoupling protein 1 (UCP1) gene requires p38 MAP kinase. J. Biol. Chem. 276, 27077–27082. 10.1074/jbc.M101049200 11369767

[B9] Carobbio S. Guenantin A. C. Samuelson I. Bahri M. Vidal-Puig A. (2019). Brown and beige fat: from molecules to physiology and pathophysiology. Biochim. Biophys. Acta Mol. Cell Biol. Lipids 1864, 37–50. 10.1016/j.bbalip.2018.05.013 29852279

[B10] Castro E. Silva T. E. O. Festuccia W. T. (2017). Critical review of beige adipocyte thermogenic activation and contribution to whole-body energy expenditure. Horm. Mol. Biol. Clin. Investig. 31. 10.1515/hmbci-2017-0042 28862985

[B11] Cheng J. T. Liu I. M. Yen S. T. Juang S. W. Liu T. P. Chan P. (2001). Stimulatory effect of D-ephedrine on beta3-adrenoceptors in adipose tissue of rats. Auton. Neurosci. 88, 1–5. 10.1016/S1566-0702(01)00225-9 11474539

[B12] Cossu S. F. Rothman S. A. Chmielewski I. L. Hsia H. H. Vogel R. L. Miller J. M. (1997). The effects of isoproterenol on the cardiac conduction system: site-specific dose dependence. J. Cardiovasc. Electrophysiol. 8, 847–853. 10.1111/j.1540-8167.1997.tb00845.x 9261710

[B13] Cui Y. C. Pan C. S. Yan L. Li L. Hu B. H. Chang X. (2017). Ginsenoside Rb1 protects against ischemia/reperfusion-induced myocardial injury *via* energy metabolism regulation mediated by RhoA signaling pathway. Sci. Rep. 7, 44579. 10.1038/srep44579 28327605 PMC5361119

[B14] Dai S. Hong Y. Xu J. Lin Y. Si Q. Gu X. (2018). Ginsenoside Rb2 promotes glucose metabolism and attenuates fat accumulation *via* AKT-dependent mechanisms. Biomed. Pharmacother. 100, 93–100. 10.1016/j.biopha.2018.01.111 29425748

[B15] Daneschvar H. L. Aronson M. D. Smetana G. W. (2016). FDA-approved anti-obesity drugs in the United States. Am. J. Med. 129, 879.e871–876. 10.1016/j.amjmed.2016.02.009 26949003

[B16] Dulloo A. G. (2011). The search for compounds that stimulate thermogenesis in obesity management: from pharmaceuticals to functional food ingredients. Obes. Rev. 12, 866–883. 10.1111/j.1467-789X.2011.00909.x 21951333

[B17] Granneman J. G. Burnazi M. Zhu Z. Schwamb L. A. (2003). White adipose tissue contributes to UCP1-independent thermogenesis. Am. J. Physiol. Endocrinol. Metab. 285, E1230–E1236. 10.1152/ajpendo.00197.2003 12954594

[B18] Guan L. Li W. Liu Z. (2002). Effect of ginsenoside-Rb1 on cardiomyocyte apoptosis after ischemia and reperfusion in rats. J. Huazhong Univ. Sci. Technol. Med. Sci. 22, 212–215. 10.1007/BF02828182 12658806

[B19] Hardie D. G. (2018). Keeping the home fires burning: AMP-activated protein kinase. J. R. Soc. Interface 15, 20170774. 10.1098/rsif.2017.0774 29343628 PMC5805978

[B20] Harms M. Seale P. (2013). Brown and beige fat: development, function and therapeutic potential. Nat. Med. 19, 1252–1263. 10.1038/nm.3361 24100998

[B21] Hong Y. Lin Y. Si Q. Yang L. Dong W. Gu X. (2019). Ginsenoside Rb2 alleviates obesity by activation of brown fat and induction of browning of white fat. Front. Endocrinol. (Lausanne) 10, 153. 10.3389/fendo.2019.00153 30930854 PMC6428988

[B22] Hossain P. Kawar B. El Nahas M. (2007). Obesity and diabetes in the developing world—a growing challenge. N. Engl. J. Med. 356, 213–215. 10.1056/NEJMp068177 17229948

[B23] Hwang J. T. Lee M. S. Kim H. J. Sung M. J. Kim H. Y. Kim M. S. (2009). Antiobesity effect of ginsenoside Rg3 involves the AMPK and PPAR-gamma signal pathways. Phytother. Res. 23, 262–266. 10.1002/ptr.2606 18844326

[B24] Inagaki T. Sakai J. Kajimura S. (2016). Transcriptional and epigenetic control of brown and beige adipose cell fate and function. Nat. Rev. Mol. Cell Biol. 17, 480–495. 10.1038/nrm.2016.62 27251423 PMC4956538

[B25] Jiang Q. S. Huang X. N. Dai Z. K. Yang G. Z. Zhou Q. X. Shi J. S. (2007). Inhibitory effect of ginsenoside Rb1 on cardiac hypertrophy induced by monocrotaline in rat. J. Ethnopharmacol. 111, 567–572. 10.1016/j.jep.2007.01.006 17374466

[B26] Jimenez M. Barbatelli G. Allevi R. Cinti S. Seydoux J. Giacobino J. P. (2003). Beta 3-adrenoceptor knockout in C57BL/6J mice depresses the occurrence of brown adipocytes in white fat. Eur. J. Biochem. 270, 699–705. 10.1046/j.1432-1033.2003.03422.x 12581209

[B27] Jung Y. Park J. Kim H. L. Sim J. E. Youn D. H. Kang J. (2018). Vanillic acid attenuates obesity via activation of the AMPK pathway and thermogenic factors *in vivo* and *in vitro* . Faseb. J. 32, 1388–1402. 10.1096/fj.201700231RR 29141998

[B28] Kim E. J. Lee H. I. Chung K. J. Noh Y. H. Ro Y. Koo J. H. (2009). The ginsenoside-Rb2 lowers cholesterol and triacylglycerol levels in 3T3-L1 adipocytes cultured under high cholesterol or fatty acids conditions. BMB Rep. 42, 194–199. 10.5483/BMBRep.2009.42.4.194 19403041

[B29] Kim S. J. Tang T. Abbott M. Viscarra J. A. Wang Y. Sul H. S. (2016). AMPK phosphorylates desnutrin/ATGL and hormone-sensitive lipase to regulate lipolysis and fatty Acid oxidation within adipose tissue. Mol. Cell. Biol. 36, 1961–1976. 10.1128/MCB.00244-16 27185873 PMC4936063

[B30] Kim S. J. Yuan H. D. Chung S. H. (2010). Ginsenoside Rg1 suppresses hepatic glucose production *via* AMP-activated protein kinase in HepG2 cells. Biol. Pharm. Bull. 33, 325–328. 10.1248/bpb.33.325 20118562

[B31] Kim S. N. Lee J. H. Shin H. Son S. H. Kim Y. S. (2009). Effects of *in vitro*–digested ginsenosides on lipid accumulation in 3T3-L1 adipocytes. Planta Med. 75, 596–601. 10.1055/s-0029-1185358 19204893

[B32] Kim W. S. Lee Y. S. Cha S. H. Jeong H. W. Choe S. S. Lee M. R. (2009). Berberine improves lipid dysregulation in obesity by controlling central and peripheral AMPK activity. Am. J. Physiol. Endocrinol. Metab. 296, E812–E819. 10.1152/ajpendo.90710.2008 19176354

[B33] Klein J. Fasshauer M. Ito M. Lowell B. B. Benito M. Kahn C. R. (1999). beta(3)-Adrenergic stimulation differentially inhibits insulin signaling and decreases insulin-induced glucose uptake in brown adipocytes. J. Biol. Chem. 274, 34795–34802. 10.1074/jbc.274.49.34795 10574950

[B34] Kong H. L. Li Z. Q. Zhao S. M. Yuan L. Miao Z. L. Liu Y. (2015). Apelin–APJ effects of ginsenoside-Rb1 depending on hypoxia-induced factor 1alpha in hypoxia neonatal cardiomyocytes. Chin. J. Integr. Med. 21, 139–146. 10.1007/s11655-014-1774-2 24893658

[B35] Kong H. L. Li Z. Q. Zhao Y. J. Zhao S. M. Zhu L. Li T. (2010). Ginsenoside Rb1 protects cardiomyocytes against CoCl2-induced apoptosis in neonatal rats by inhibiting mitochondria permeability transition pore opening. Acta Pharmacol. Sin. 31, 687–695. 10.1038/aps.2010.52 20523339 PMC4002974

[B36] Kwan H. Y. Wu J. Su T. Chao X. J. Liu B. Fu X. (2017). Cinnamon induces browning in subcutaneous adipocytes. Sci. Rep. 7, 2447. 10.1038/s41598-017-02263-5 28550279 PMC5446408

[B37] Lee H. M. Lee O. H. Kim K. J. Lee B. Y. (2012). Ginsenoside Rg1 promotes glucose uptake through activated AMPK pathway in insulin-resistant muscle cells. Phytother. Res. 26, 1017–1022. 10.1002/ptr.3686 22170817

[B38] Lee J. E. Lee H. Kim M. H. Yang W. M. (2019). Osteogenic effects of *Phlomis umbrosa via* up-regulation of Runx2 in osteoporosis. Biomed. Rep. 10, 17–22. 10.3892/br.2018.1172 30588298 PMC6299205

[B39] Lee J. Y. Takahashi N. Yasubuchi M. Kim Y. I. Hashizaki H. Kim M. J. (2012). Triiodothyronine induces UCP-1 expression and mitochondrial biogenesis in human adipocytes. Am. J. Physiol. Cell Physiol. 302, C463–C472. 10.1152/ajpcell.00010.2011 22075692

[B40] Lee J. B. Yoon S. J. Lee S. H. Lee M. S. Jung H. Kim T. D. (2017). Ginsenoside Rg3 ameliorated HFD-induced hepatic steatosis through downregulation of STAT5-PPARγ. J. Endocrinol. 235, 223–235. 10.1530/JOE-17-0233 29042402

[B41] Lee K. Seo Y. J. Song J. H. Lee B. Y. (2018). Ginsenoside Rg1 promotes browning by inducing UCP1 expression and mitochondrial activity in 3T3-L1 and subcutaneous white adipocytes. J. Ginseng Res. xxx, 1–11. 10.1016/j.jgr.2018.07.005 PMC6823768 31695565

[B42] Lee O. H. Lee H. H. Kim J. H. Lee B. Y. (2011). Effect of ginsenosides Rg3 and Re on glucose transport in mature 3T3-L1 adipocytes. Phytother. Res. 25, 768–773. 10.1002/ptr.3322 21520470

[B43] Li G. Qian W. Zhao C. (2016). Analyzing the anti–ischemia-reperfusion injury effects of ginsenoside Rb1 mediated through the inhibition of p38alpha MAPK. Can. J. Physiol. Pharmacol. 94, 97–103. 10.1139/cjpp-2014-0164 26550918

[B44] Li J. Shao Z. H. Xie J. T. Wang C. Z. Ramachandran S. Yin J. J. (2012). The effects of ginsenoside Rb1 on JNK in oxidative injury in cardiomyocytes. Arch. Pharm. Res. 35, 1259–1267. 10.1007/s12272-012-0717-3 22864749 PMC3415887

[B45] Li J. B. Zhang R. Han X. Piao C. L. (2018). Ginsenoside Rg1 inhibits dietary-induced obesity and improves obesity-related glucose metabolic disorders. Braz. J. Med. Biol. Res. 51, e7139. 10.1590/1414-431x20177139 29513799 PMC5856439

[B46] Li P. Zhu Z. Lu Y. Granneman J. G. (2005). Metabolic and cellular plasticity in white adipose tissue II: role of peroxisome proliferator–activated receptor-alpha. Am. J. Physiol. Endocrinol. Metab. 289, E617–E626. 10.1152/ajpendo.00010.2005 15941786

[B47] Li Q. Y. Chen L. Fu W. H. Li Z. D. Wang B. Shi X. J. (2011). Ginsenoside Rb1 inhibits proliferation and inflammatory responses in rat aortic smooth muscle cells. J. Agric. Food Chem. 59, 6312–6318. 10.1021/jf200424k 21524054

[B48] Lin N. Cai D. L. Jin D. Chen Y. Shi J. J. (2014). Ginseng panaxoside Rb1 reduces body weight in diet-induced obese mice. Cell Biochem. Biophys. 68, 189–194. 10.1007/s12013-013-9688-3 23733675

[B49] Liu H. Wang J. Liu M. Zhao H. Yaqoob S. Zheng M. (2018). Antiobesity effects of ginsenoside Rg1 on 3T3-L1 preadipocytes and high fat diet–induced obese mice mediated by AMPK. Nutrients 27, E830. 10.3390/nu10070830 PMC6073290 29954059

[B50] Lowell B. B. Spiegelman B. M. (2000). Towards a molecular understanding of adaptive thermogenesis. Nature 404, 652–660. 10.1038/35007527 10766252

[B51] Lü J. M. Yao Q. Chen C. (2009). Ginseng compounds: an update on their molecular mechanisms and medical applications. Curr. Vasc. Pharmacol. 7, 293–302. 10.2174/157016109788340767 19601854 PMC2928028

[B52] Luo X. Jia R. Zhang Q. Sun B. Yan J. (2016). Cold-induced browning dynamically alters the expression profiles of inflammatory adipokines with tissue specificity in mice. Int. J. Mol. Sci. 17, E795. 10.3390/ijms17050795 27223282 PMC4881611

[B53] Michan S. Sinclair D. (2007). Sirtuins in mammals: insights into their biological function. Biochem. J. 404, 1–13. 10.1042/BJ20070140 17447894 PMC2753453

[B54] Mu Q. Fang X. Li X. Zhao D. Mo F. Jiang G. (2015). Ginsenoside Rb1 promotes browning through regulation of PPARgamma in 3T3-L1 adipocytes. Biochem. Biophys. Res. Commun. 466, 530–535. 10.1016/j.bbrc.2015.09.064 26381176

[B55] Oakhill J. S. Scott J. W. Kemp B. E. (2012). AMPK functions as an adenylate charge–regulated protein kinase. Trends Endocrinol. Metab. 23, 125–132. 10.1016/j.tem.2011.12.006 22284532

[B56] Park S. Ahn I. S. Kwon D. Y. Ko B. S. Jun W. K. (2008). Ginsenosides Rb1 and Rg1 suppress triglyceride accumulation in 3T3-L1 adipocytes and enhance beta-cell insulin secretion and viability in Min6 cells *via* PKA-dependent pathways. Biosci. Biotechnol. Biochem. 72, 2815–2823. 10.1271/bbb.80205 18997435

[B57] Qiao L. Zhang X. Liu M. Liu X. Dong M. Cheng J. (2017). Ginsenoside Rb1 enhances atherosclerotic plaque stability by improving autophagy and lipid metabolism in macrophage foam cells. Front. Pharmacol. 8, 727. 10.3389/fphar.2017.00727 29114222 PMC5660703

[B58] Rachid T. L. Penna-De-Carvalho A. Bringhenti I. Aguila M. B. Mandarim-De-Lacerda C. A. Souza-Mello V. (2015). Fenofibrate (PPARalpha agonist) induces beige cell formation in subcutaneous white adipose tissue from diet-induced male obese mice. Mol. Cell Endocrinol. 402, 86–94. 10.1016/j.mce.2014.12.027 25576856

[B59] Ruderman N. B. Xu X. J. Nelson L. Cacicedo J. M. Saha A. K. Lan F. (2010). AMPK and SIRT1: a long-standing partnership? Am. J. Physiol. Endocrinol. Metab. 298, E751–E760. 10.1152/ajpendo.00745.2009 20103737 PMC2853213

[B60] Sears M. R. (2002). Adverse effects of beta-agonists. J. Allergy Clin. Immunol. 110, S322–S328. 10.1067/mai.2002.129966 12464943

[B61] Shang W. Yang Y. Jiang B. Jin H. Zhou L. Liu S. (2007). Ginsenoside Rb1 promotes adipogenesis in 3T3-L1 cells by enhancing PPARgamma2 and C/EBPalpha gene expression. Life Sci. 80, 618–625. 10.1016/j.lfs.2006.10.021 17129589

[B62] Shen L. Xiong Y. Wang D. Q. Howles P. Basford J. E. Wang J. (2013). Ginsenoside Rb1 reduces fatty liver by activating AMP-activated protein kinase in obese rats. J. Lipid Res. 54, 1430–1438. 10.1194/jlr.M035907 23434611 PMC3622335

[B63] Shi T. Fan G. Q. Xiao S. D. (2010). SIRT3 reduces lipid accumulation *via* AMPK activation in human hepatic cells. J. Dig. Dis. 11, 55–62. 10.1111/j.1751-2980.2009.00416.x 20132432

[B64] Smith R. E. (1961). Thermogenic activity of the hibernating gland in the cold-acclimated rat. Physiologist 4, 113.

[B65] Steensels S. Ersoy B. A. (2019). Fatty acid activation in thermogenic adipose tissue. Biochim. Biophys. Acta Mol. Cell Biol. Lipids 1864, 79–90. 10.1016/j.bbalip.2018.05.008 29793055

[B66] Steinberg G. R. Carling D. (2019). AMP-activated protein kinase: the current landscape for drug development. Nat. Rev. Drug. Discov. 18, 527–551. 10.1038/s41573-019-0019-2 30867601

[B67] Virtanen K. A. (2016). The rediscovery of BAT in adult humans using imaging. Best Pract. Res. Clin. Endocrinol. Metab. 30, 471–477. 10.1016/j.beem.2016.09.001 27697208

[B68] Vitali A. Murano I. Zingaretti M. C. Frontini A. Ricquier D. Cinti S. (2012). The adipose organ of obesity-prone C57BL/6J mice is composed of mixed white and brown adipocytes. J. Lipid Res. 53, 619–629. 10.1194/jlr.M018846 22271685 PMC3307639

[B69] Wang J. Liu R. Wang F. Hong J. Li X. Chen M. (2013). Ablation of LGR4 promotes energy expenditure by driving white-to-brown fat switch. Nat. Cell. Biol. 15, 1455–1463. 10.1038/ncb2867 24212090

[B70] Wang S. Liang X. Yang Q. Fu X. Rogers C. J. Zhu M. (2015). Resveratrol induces brown-like adipocyte formation in white fat through activation of AMP-activated protein kinase (AMPK) alpha1. Int. J. Obes. (Lond.) 39, 967–976. 10.1038/ijo.2015.23 25761413 PMC4575949

[B71] Wang Z. Li M. Wu W. K. Tan H. M. Geng D. F. (2008). Ginsenoside Rb1 preconditioning protects against myocardial infarction after regional ischemia and reperfusion by activation of phosphatidylinositol-3-kinase signal transduction. Cardiovasc. Drugs Ther. 22, 443–452. 10.1007/s10557-008-6129-4 18679782

[B72] WHO (2018). WHO media centre fact sheets: obesity and overweight [Online]. http://apps.who.int/mediacentre/factsheets/fs311/en/index.html [Accessed April 2019].

[B73] Wu J. Bostrom P. Sparks L. M. Ye L. Choi J. H. Giang A. H. (2012). Beige adipocytes are a distinct type of thermogenic fat cell in mouse and human. Cell 150, 366–376. 10.1016/j.cell.2012.05.016 22796012 PMC3402601

[B74] Wu Y. Xia Z. Y. Dou J. Zhang L. Xu J. J. Zhao B. (2011). Protective effect of ginsenoside Rb1 against myocardial ischemia/reperfusion injury in streptozotocin-induced diabetic rats. Mol. Biol. Rep. 38, 4327–4335. 10.1007/s11033-010-0558-4 21113666

[B75] Xia R. Zhao B. Wu Y. Hou J. B. Zhang L. Xu J. J. (2011). Ginsenoside Rb1 preconditioning enhances eNOS expression and attenuates myocardial ischemia/reperfusion injury in diabetic rats. J. Biomed. Biotechnol. 2011, 767930. 10.1155/2011/767930 22013385 PMC3196378

[B76] Xiong Y. Shen L. Liu K. J. Tso P. Xiong Y. Wang G. (2010). Antiobesity and antihyperglycemic effects of ginsenoside Rb1 in rats. Diabetes 59, 2505–2512. 10.2337/db10-0315 20682695 PMC3279544

[B77] Yan X. Liu J. Wu H. Liu Y. Zheng S. Zhang C. (2016). Impact of miR-208 and its target gene nemo-like kinase on the protective effect of ginsenoside Rb1 in hypoxia/ischemia injuried cardiomyocytes. Cell Physiol. Biochem. 39, 1187–1195. 10.1159/000447825 27577116

[B78] Yan X. Xue J. Wu H. Wang S. Liu Y. Zheng S. (2015). Ginsenoside-Rb1 protects hypoxic- and ischemic-damaged cardiomyocytes by regulating expression of miRNAs. Evid. Based Complement Alternat. Med. 2015, 171306. 10.1155/2015/171306 26074986 PMC4449925

[B79] Yao X. Shan S. Zhang Y. Ying H. (2011). Recent progress in the study of brown adipose tissue. Cell Biosci. 1, 35. 10.1186/2045-3701-1-35 22035495 PMC3219668

[B80] Yu X. Ye L. Zhang H. Zhao J. Wang G. Guo C. (2015). Ginsenoside Rb1 ameliorates liver fat accumulation by upregulating perilipin expression in adipose tissue of db/db obese mice. J. Ginseng Res. 39, 199–205. 10.1016/j.jgr.2014.11.004 26199550 PMC4506369

[B81] Zhang L. Zhang L. Wang X. Si H. (2017). Anti-adipogenic effects and mechanisms of ginsenoside Rg3 in pre-adipocytes and obese mice. Front. Pharmacol. 8, 113. 10.3389/fphar.2017.00113 28337143 PMC5340763

[B82] Zhang X. Liu M. H. Qiao L. Zhang X. Y. Liu X. L. Dong M. (2018). Ginsenoside Rb1 enhances atherosclerotic plaque stability by skewing macrophages to the M2 phenotype. J. Cell Mol. Med. 22, 409–416. 10.1111/jcmm.13329 28944992 PMC5742675

[B83] Zhang X. J. He C. Tian K. Li P. Su H. Wan J. B. (2015). Ginsenoside Rb1 attenuates angiotensin II–induced abdominal aortic aneurysm through inactivation of the JNK and p38 signaling pathways. Vascul. Pharmacol. 73, 86–95. 10.1016/j.vph.2015.04.003 25912763

[B84] Zheng Q. Bao X. Y. Zhu P. C. Tong Q. Zheng G. Q. Wang Y. (2017a). Ginsenoside Rb1 for myocardial ischemia/reperfusion injury: preclinical evidence and possible mechanisms. Oxid. Med. Cell Longev. 2017, 6313625. 10.1155/2017/6313625 29430282 PMC5753014

[B85] Zheng X. Wang S. Zou X. Jing Y. Yang R. Li S. (2017b). Ginsenoside Rb1 improves cardiac function and remodeling in heart failure. Exp. Anim. 66, 217–228. 10.1538/expanim.16-0121 28367863 PMC5543242

[B86] Zhou P. Lu S. Luo Y. Wang S. Yang K. Zhai Y. (2017). Attenuation of TNF-alpha–induced inflammatory injury in endothelial cells by ginsenoside Rb1 *via* inhibiting NF-kappaB, JNK and p38 signaling pathways. Front. Pharmacol. 8, 464. 10.3389/fphar.2017.00464 28824425 PMC5540891

[B87] Zhou P. Xie W. Sun Y. Dai Z. Li G. Sun G. (2019). Ginsenoside Rb1 and mitochondria: a short review of the literature. Mol. Cell. Probes 43, 1–5. 10.1016/j.mcp.2018.12.001 30529056

[B88] Zhu L. Luan X. Dou D. Huang L. (2019). Comparative Analysis of Ginsenosides and Oligosaccharides in White Ginseng (WG), red Ginseng (RG) and Black Ginseng (BG). J. Chromatogr. Sci. 57, 403–410. 10.1093/chromsci/bmz004 30839052

