# Supplementary material for: Ginsenoside Rb1 Induces Beta 3 Adrenergic Receptor–Dependent Lipolysis and Thermogenesis in 3T3-L1 Adipocytes and db/db Mice
Source: Front Pharmacol. 2019 Oct 15;10:1154. doi: 10.3389/fphar.2019.01154 (PMC6803469; doi:10.3389/fphar.2019.01154)
Supplement: Supplementary file 1 [file Image_1.pdf]

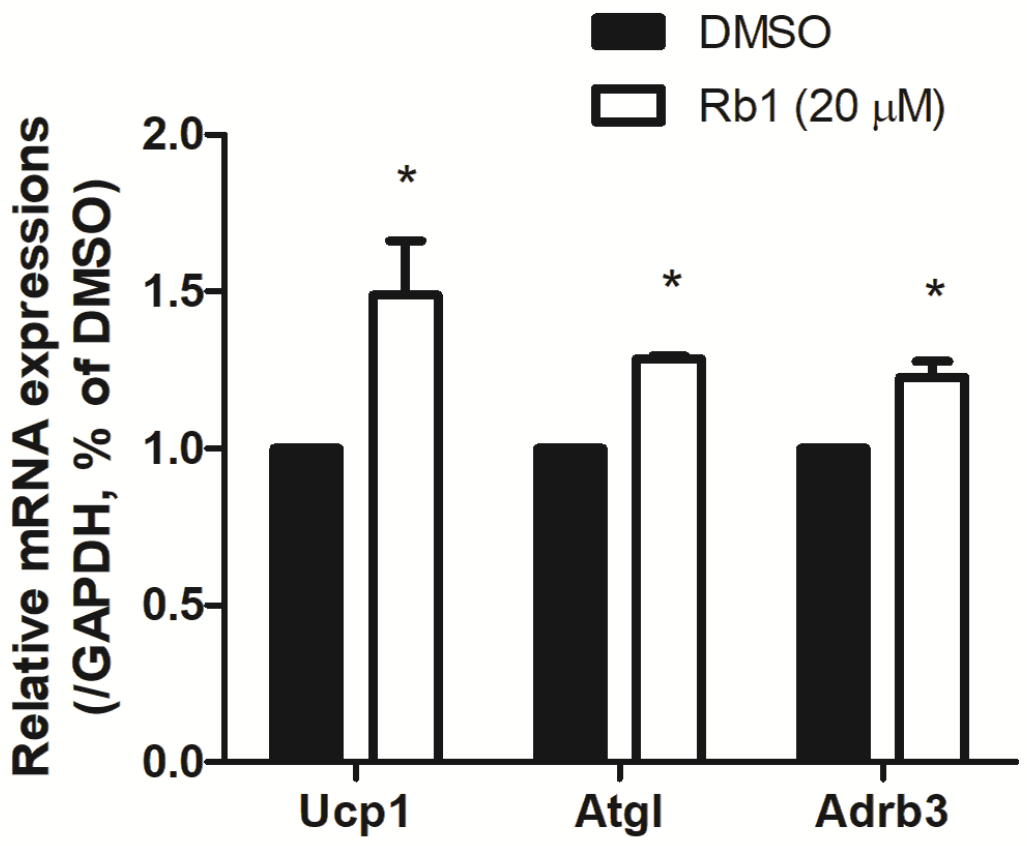

SUPPLEMENTARY FIGURE S1: Effect of Rb1 in beige-induced SVF cells. mRNA levels of *Ucp1*, *Atgl* and *Adrb3* were analyzed in Rb1-treated beige-induced SVF cells. Relative mRNA level of each gene was normalized to level of *Gapdh*. Data are expressed as mean ± S.E.M. of three or more experiments. $^{*}p < 0.05$ vs. DMSO-treated beige-induced SVF cells. Rb1, ginsenoside Rb1.

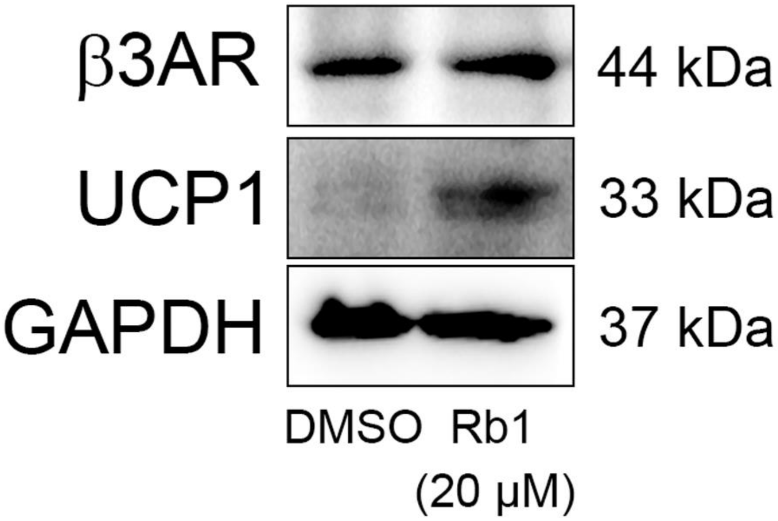

SUPPLEMENTARY FIGURE S2: Effect of Rb1 in white-induced hAMSCs. Protein levels of β3AR and UCP1 were measured in Rb1-treated white-induced hAMSCs. DMSO was used as vehicle. Rb1, ginsenoside Rb1.

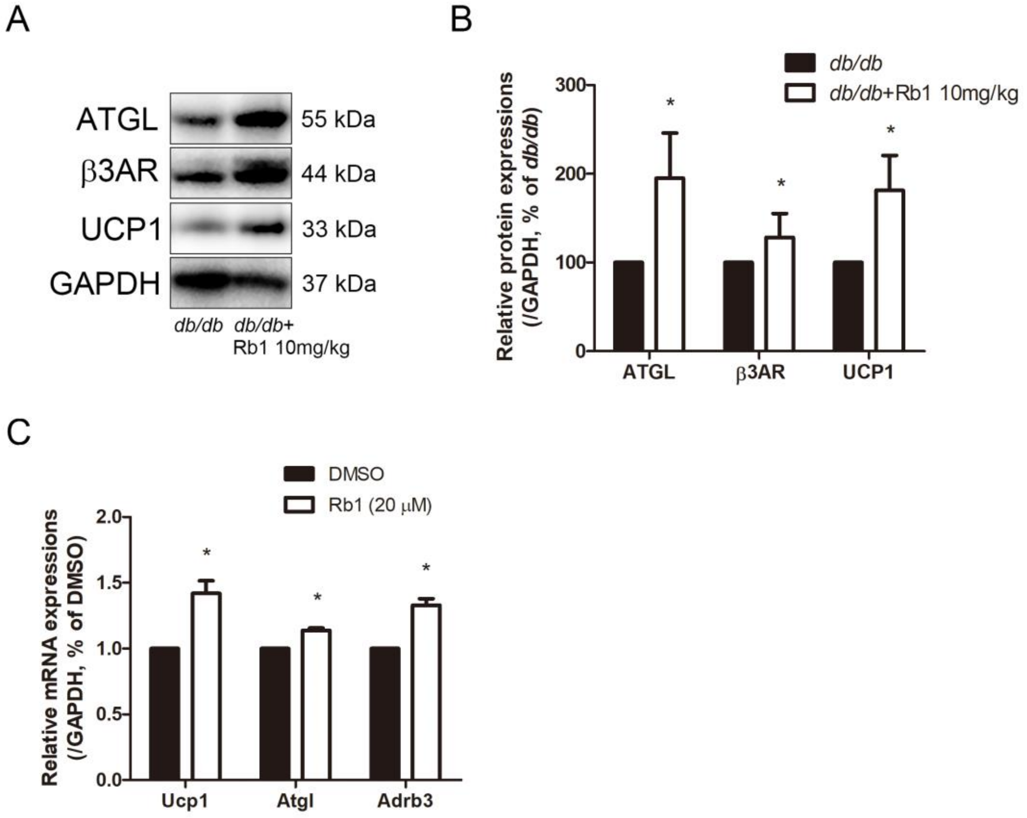

SUPPLEMENTARY FIGURE S3: Effect of Rb1 in BAT of *db/db* mice and primary cultured brown adipocytes. (A) Protein levels of ATGL, β3AR and UCP1 were measured in BAT of Rb1-treated *db/db* mice. (B) Relative expression levels of UCP1, ATGL and β3AR were quantified. Expressions of UCP1, ATGL and β3AR were normalized against GAPDH. (C) mRNA levels of *Ucp1*, *Atgl* and *Adbr3* were analyzed analyzed in Rb1-treated primary cultured brown adipocytes. Relative mRNA level of each gene was normalized to level of *Gapdh*. PBS was used as vehicle in vivo experiments; DMSO was used as vehicle in vitro experiments. Data are expressed as mean ± S.E.M. of three or more experiments. $^{*}p < 0.05$ vs. vehicle-treated *db/db* mice or primary cultured brown adipocytes. Rb1, ginsenoside Rb1.
